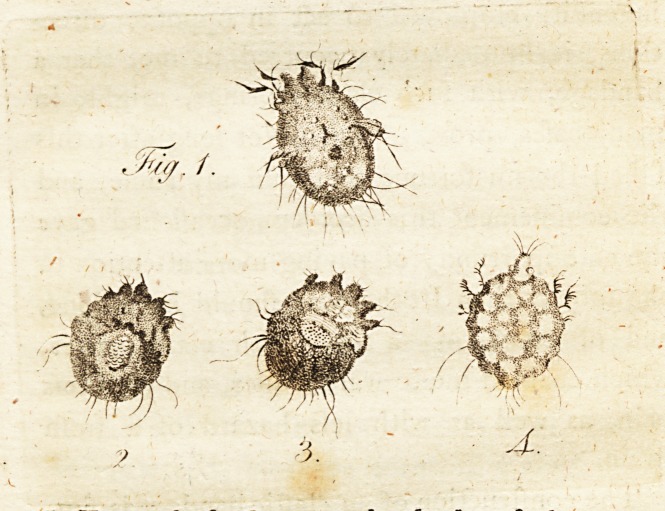# An Account of the Insect Found in the Itch. From a Work Lately Published, in German, on the Etiology of That Disease

**Published:** 1788

**Authors:** J. E. Wichmann

**Affiliations:** Physician to His Majesty at Hanover, and Member of the Royal Society of Sciences at Goettingen, &c.


### II. *An Account of the Insect found in the Itch. From a Work lately published, in German, on the Etiology of that Disease *, by* J. E. Wichmann, *M. D. Physician to His Majesty at Hanover, and Member of the Royal Society of Sciences at Goettingen, &c.*

THAT the itch is simply a local affection of the skin, occasioned by animalcula, has been a pretty general opinion in this country, since the description given by the late Dr. Mead † of the insect found in this disease by Bonomo. But, of those who have adopted this idea, very few, probably, have had an opportunity of convincing themselves, by their own observation, how far it is founded in truth; and it is certain there are many who still doubt, and even deny, the existence of the insect in question. It was the difference of opinion that prevails on this head, and the doubts he himself

* Aetiologie der Kraetze; von Johann Ernst Wichmann, d. A. D. Koenigl. Grossbritt. Hofmedicus zu Hannover, Mitgliede der Koenigl. Gesellschaft der Wissenschaften zu Goettingen, und der Gesellschaft Naturforschender Freunde zu Berlin. 8vo. Hannover, 1786.

† Philosophical Transactions, Vol. XXIII. for the year 1702, page 1296.

had had concerning it, that induced the author of the work now before us to direct his attention to this subject.

The first account he met with of these insects was by Moufet, whose description of the *Syrones*, in his *Theatrum Insectorum* *, he supposes to be applicable to the itch insect. Of the Syro Moufet says, " Animalculum " est omnium minutissimum, solens innasci " caseo, & ceræ inveteratis, & cuti item hu- " manæ...... Anglice *mites*, in caseo, foliis, " ligno arido, atque cera; sed in homine " *wheale wormes* dicuntur, & Germanice *Seu-* " *ren*. Ita sub cute habitat, ut actis cuniculis " pruritum maximum loco ingeneret, præci- " pue manibus, vel aliis partibus affectis & igni " admotis. Extractus acu, & super ungue po- " situs, movet se, si solis etiam calore adjuve- " tur....... Hoc obiter est observandum, Sy- " rones istos non in ipsis pustulis, sed prope " habitare. Illorum quippe proprium est non " longe residere ab humore aqueo in vesicula " vel pustula collecto: quo absumpto, vel ex- " iccato, brevi omnes intereunt. Neque Sy-

* Page 266.

rones

" rones isti sunt de pediculorum genere; nam " illi extra cutem vivunt, hi vero non."

In the same work also he found the following quotation, relative to the Syrones, from the writings of an Arabian physician, who flourished in the twelfth century: " *Syrones* (inquit " Abinzoar), *Assoalat* & *Assoab* dicti, sunt pe- " dicelli subter manuum crurumque & pedum " cutem serpentes, & pustulas ibidem exci- " tantes aquâ plenas: tam parva animalcula, " ut vix visu perspicaci discerni valeant *."

As Moufet, whose work was published in 1634, mentions the name those insects go by among the Germans, our author's curiosity led him to examine the German writers on insects before that period, but without being able to find any thing on this subject. In a work †, however, by Hauptmanns, a physician at Dresden, published twenty years after Moufet's, he unexpectedly met with something relative to this matter. This writer mentions the animalcula he had found in the itch, and which, he says, are called *Acari* or *Cyrones*, and by the

* Theatrum Iusectorum, page 266.

† Uhralten Wolkensteinischen Warmen Bad und Wasser schatze. 8vo. Dresden, 1654.

Germans

Germans *Rietliesen*. He obferves, that fo far as he had examined them with a microfcope, they feemed to agree with the infects which are found in cheefe. Our author fuppofes this writer to have been the firft who has given a figure of the itch infect; but this figure is inaccurate, and the defcription, like the preceding accounts of it, obfcure; fo that the exiftence of the infect can hardly be faid to have been clearly and fatisfactorily afcertained till the time of Bonomo.

The Italian original of the letter * from Bonomo to Redi on this fubject, printed at Florence in 1683, is now extremely fcarce; but a Latin tranflation of it, by Lanzoni, may be found in the Mifcell. Natur. Curiof. †. This

* Offervazioni intorno a pelicelli del corpo umano dal G. Cof. Bonomo, e da lui con altre offervazioni fcritte in una lettera al Fr. Redi.

† Obfervationes circa humani Corporis Teredinem, a Cl. Joh. Cofmo Bonomo, practico infigniffimo Liburni, una cum aliis epiftolica hac in exercitatione ad Eoos penes Hefperiofq. famigeratiffimum, illuftr. Francifcum Redum, Italico fermone anno 1687 confcriptæ & Florentiæ typis impreffæ, nunc vero Latinitate donatæ a Jofepho Lanzono, Acad. Cur.— Vid. Append. ad annum Decimum Decuriæ 2. Ephem. Med. Phyf. Nat. Cur. 4to. Norimbergæ, 1692.

discovery, discovery, however, did not seem to excite much attention till Dr. Mead * gave an account of

* Dr. Wichmann has inserted in his work the whole of Dr. Mead's paper on this subject; but to reprint it here would be superfluous, as it may be found not only in the Philosophical Transactions, but also in Dr. Mead's works, and in Mihles's Medical Essays.—It may not be improper, however, to observe, that Dr. Mead, by omitting the beginning of Bonomo's letter to Redi, has not fully stated the circumstances that led to the discovery of the insect in question, and has given to Bonomo the credit of observations for which we find Bonomo acknowledging himself indebted to one of his friends, whom he names. As the passage relative to this matter, in Bonomo's letter, is curious, we shall transcribe it from the Latin translation by Lanzoni. It is as follows:

" Casu fortuitove se mihi legendum obtulit in celebri *Vocabolario dell' Academia della Crusca* ab hujus compilatoribus asseri Teredinem, qua ut plurimum scabie infectorum cutis scalet, in perexiguis, ac minutulis animalculis consistere; ecce ipsissima Vocabularii verba: *Pellicello i un picolissimo Bacolino, il quale si genera a Rognosi in pelle e rodendo cagiona un' acutissimo pizzicore.* Idem sentire postmodum observavi Joseph. Laurentium in sua Amalthea, dum scripsit: *Acarus. Teredo. Vermiculus exiguus subcutaneus rodens. Pidicello.* et *Lit. T. Teredo. Vermis in ligno nascens: Caries. Item acarus rodens carnem sub cute; Pidicello.* His itaque sic lectitatis, iterata, sedulaque experientia scrutandi prurigine tactus sum, an dictæ Teredines animalcula vere sint, serioque consului eruditiss. Hyacin-

" thum of it in the Philofophical Tranfactions; and even fince that time many of the writers who have defcribed this infect have contented themfelves with copying the obfervations of Bonomo, without examining the matter themfelves: our author particularly mentions Bonanni *, Schwiebe †, and Baker ‡, whofe defcriptions are all taken from this fource. Even fome of thofe, our author remarks, who have

" thum Ceftonium, ejus in experiendo probatæ fedulitatis; " quæ multo ante tibi vir cl. innotuit. Multoties ergo obfervaffe mihi conftanter affeveravit, mulierculas propriis e fcabiofis filiolis acus extremitate nefcio quid educere, quod in " lævæ manus pollicis ungue, alterius manus pollicis ungue " compreffum, in ipfa compreffione aliquem parvum fonum " facere videtur, hoc autem educi a minutioribus tuberculis " fcabiofis, perfecta nondum fanie fcatentibus, vel ut vocitant immaturis; mutua quod itidem charitate inter remiges, " & mancipia Balnei Liburnenfis, fi fcabies infeftaret, fieri " adnotavit. Inde fubdidit non fibi tamen certo conftare, an " Teredines e vermiculorum cenfu forent, prompte tamen de " eo certiorem fe reddendi occafionem daturam, multis experimentis in fcabiofo quopiam, quo autopfia infallibili in affirmativam, vel negativam partem declinandum doceremur." — Editor.

* Obferv. circa vivent. Romæ, 1699.

† Differt. de pruritu exanthematum ab acaris. Lipfiæ, 1722.

‡ Microfcope made eafy. 8vo. London, 1743.

 acquired acquired reputation in the present century by microscopical discoveries, as Leeuwenhoeck, Reaumur, and Swammerdam, have either not thought it worth their while to examine this matter, or, like many learned men now living, were perhaps unable, for want of the necessary dexterity, to find these insects. It is certain, he adds, that Leeuwenhoeck *, although he has accurately described the acari of meal, has totally omitted those found in the itch.

At length the attention of physicians and naturalists was directed afresh to this subject by the celebrated Linnæus †; and the itch insect was almost generally admitted even by those who had not seen the insect, but who relied on the authority of that great naturalist for its existence.

After giving an account of the discovery of these insects, our author proceeds to consider more particularly their natural history. The genus (*Acarus*) to which they belong is, he observes, very numerous, and its different species have not all of them been accurately determined. He confines his inquiries, however,

* Arcana Naturæ detect. 4to. 1722. Epist. 77, p. 356.

† Exanthemata viva. 4to. Upsal. 1757.

to to the two ſpecies which are found in meal and in the itch.

That there ſhould be confuſion in determining theſe ſpecies, he thinks, will not be wondered at, when we are told that even at the preſent day naturaliſts differ concerning their figure, and diſpute whether what one ſees on the head of theſe minute animals are to be conſidered as antennæ or feet. Thus, of many naturaliſts, to name only a few of rank, Linnæus * has only *tentacula*; Schæffer † has *antennæ pediformes articulatæ*; while Baron de Geer ‡ expreſſly ſays they have no antennæ, but two arms, with joints, which reſemble thoſe of ſpiders, who have likewiſe no antennæ.

Another ſource of confuſion in the arrangement of theſe inſects has ariſen, our author thinks, from the ambiguity of the generical character, which depends on the number of eight feet, whereas many have obſerved only ſix. Baron de Geer, however, has explained this by ſhewing that in the young acari farinæ the eighth

* Syſtem. Natur. Edit. XII.

† Elementa Entomologiæ. 4to. 1766.

‡ Memoires pour ſervir a l'Hiſtoire des Inſectes. 1778. Tom. VII. p. 85.

pair is wanting. This obſervation, however, Dr. Wichmann remarks, is not new, having been made long ago by Leeuwenhoeck *. He himſelf, he tells us, as well as Mr. Goetze †, in examining the acari of meal, has frequently found ſome with ſix, and others with eight, feet; but in thoſe of cheeſe he has uniformly found eight.

Linnæus, our author obſerves, in his diſſertation already quoted, entitled *Exanthemata viva*, aſſerts, that nurſes, when they ſprinkle children under the axillæ, &c. with flour that contains acari, give them the itch; and from this infers that the acarus of meal and that of the itch are of the ſame ſpecies ‡. But Dr. Wichmann contends, and we believe very juſtly, that the erup-

* Arcan. Natur. 4to. 1722. p. 356.

† Abhandl. aus der Inſectologie, p. 333.

‡ "Caſeum vel farinam, diu de loco non motam, multa "horum millia alere, non raro obſervamus; hinc evenit, ut, "quum nutrices loco Pollinis Lycopodii, Florum Zinci, &c. "infantes intertrigine laborantes *farina* frumenti conſpergant, "inguina & axillæ, eadem adſperſæ, in ſcabiem effloreſcant; "quod malum, ſæpius curatum, identidem rediit, quoties "farinæ adſperſio iterata fuerit, alioſque infecit infantes. "Hinc Farinæ & Scabiei Acaros unam conſtituere candemque "ſpeciem concludimus."

tion tion which is ſometimes excited in children by this means is very different from the true itch, and that it ſoon diſappears without any aſſiſtance from medicine. He obſerves alſo that in the Fauna Suecica *, Linnæus, although he has there accurately deſcribed the inſect found in the itch, has confounded it with the acarus of meal; and that ſtill more lately, in the twelfth and laſt edition of his Syſtema Naturæ, he had ſo little altered his opinion, that, after deſcribing the Acarus Siro, he expreſſly adds, "Inter "Sirones farinæ, Scabiei . . . vix etiamnum reperi alias differentias, quam a loco petitas †."

The

* Page 482.

† We think it right to obſerve here, that the ſeeming confuſion on this ſubject, in the writings of Linnæus, appears to have ariſen from an opinion he had adopted, of the exiſtence of more than one ſpecies of itch; for beſides the itch which he imagined might be excited by the *Acarus Siro*, or that ſpecies which is found in meal, he ſuppoſed that in another and more inveterate kind of itch, the *Scabies ferina*, the diſeaſe is occaſioned by a different ſpecies of *Acarus*, the *Acarus exulcerans*, which he has deſcribed with his uſual accuracy, and which is indiſputably the true itch inſect. This he no where confounds with the *A. Siro*, but expreſſly ſays it is a diſtinct ſpecies. Thus, in the Diſſertation *(Exanthemata viva)* juſt now

The German tranflator and commentator of Linnæus, Profeffor Muller, has taken occafion * to obferve, however, that a difference exifts between the acari of cheefe and thofe of meal, as well as between the latter and thofe of the itch; but feveral medical writers, our author remarks, (and particularly Rofenftein, in his Treatife on the Difeafes of Children) relying on the authority of Linnæus, that the fame animalcula are found in meal as in the itch, have afferted that flour, in which there are acari, is capable of communicating this difeafe.. To this confufion of fpecies our aunow quoted, we find him obferving, that "In fcabie ferina "acari ægrius inveniuntur; exemtos vero, *aliam effe fpeciem* "(acarus exulcerans) & pedibus quatuor pofticis, corpore "duplo longioribus, diftinctos." And again, in his Syftem. Natur. immediately after the *Acarus Siro*, he places the "*Acarus exulcerans*, pedibus longiffimis fetaceis; anticis "duobus brevibus;" and adds, "habitat in fcabie ferina." A fimilar divifion of the itch, into a mild fpecies and one more virulent, was made by the ancients. Thus Celfus, (de Medicin. lib. v. cap. 28.) in treating of the itch (*fcabies*), obferves, that "Quo afperior eft, quoque prurit magis, eo dif-"ficilius tollitur. Itaque eam, quæ talis eft, ἄγριαν Græci "appellant, id eft *feram*." — EDITOR.

* Linné, Natur-Syftem. Nurnberg, 1775. Part. V. p. 1050.

thor thor attributes an aſſertion by Profeſſor Murray *, in his, in other reſpects, judicious account of the itch, viz. that, previous to any appearance of puſtules, there is always a foulneſs of the juices, and that when this foulneſs has got to a certain height, the acari of cheeſe or meal are induced to ſeek a nidus in the ſkin; and of courſe he muſt ſuppoſe theſe to be of the ſame ſpecies as thoſe of the itch.

Profeſſor Pallas alſo, Dr. Wichmann obſerves, has omitted to diſtinguiſh theſe inſects properly, as he ſays, "Acarus ſcabiei, acaro farinæ "eſt conſanguineus †." But Baron de Geer, he acknowledges, has very accurately diſcriminated theſe ſpecies, and ſhewn that the *acarus domeſticus*, (or that ſpecies which is found in cheeſe, &c.) the *acarus farinæ*, and the *acaras ſcabiei*, are all very different from each other. Of the ſecond of theſe ſpecies, he ſays, "Acarus (farinæ) oblongus albus, ca-"pite ruſeſcente, pedibus conicis craſſioribus "æqualibus;" and of the laſt, or itch inſect, "Acarus (ſcabiei) ſubrotundus albus, pedibus

* De Vermibus in Lepra obviis. 4to. Goetting. 1769. p. 9.

† Diſſ. de Inſeſtis viventibus. 4to. 1760. p. 2.

"ruſeſcen-

" rufefcentibus brevibus; pofticis quatuor feta
" longiffima, plantis quatuor anticis fiftulatis
" capitulo terminatis *."

In fpeaking of the manner of finding thefe infects in the itch, our author obferves, that the failure of many who have fought for them has been owing to their having expected to meet with them in the larger veficles that contain a yellowifh fluid, like pus; in thefe, however, he tells us, he has never found them, but in thofe puftules only which are recent, and contain only a watery fluid: we muft, therefore, he obferves, not expect to find them in the fame proportionate number in patients, who, for many months, have been afflicted with the difeafe, as in thofe in whom its appearance is recent, and where it is confined to the fingers or wrifts. The caufe of this difference with refpect to the puftules, he conjectures, may be owing to the death of the infect after it has depofited its eggs.

A fmall tranfparent veficle being found, a very minute, white point, diftinct from the furrounding fluid, may be difcovered, and very

* Mem. pour fervir a l'Hiftoire des Infectes. Tom. VII. p. 94.

often often even without the affiftance of a glafs; this is the infect, which may eafily be taken out on the point of a needle or penknife, and when placed on a green cloth, may be feen much more diftinctly, and obferved to move *.

The author remarks, that even before fuch a tranfparent veficle is formed, we may often dif-cover traces of the infect on the fingers or hands, in a reddifh ftreak or furrow, which is occafioned by the acarus; and he adds, that it is even more ufual to find it in thefe furrows than in the puftules themfelves. He tells us,

* Fabricius (Faun. Groenland. p. 221) has mentioned the dexterity of the Groenlanders in extracting this infect. "Ha-"bitat," fays he, "in veficula fcabiei Groenlandorum, qui "illum acu eximere fcientes, mihi miranti, ut vivum animal "incedentum oftenderunt." — Linnæus, in defcribing it, (Faun. Suecic. 1194) fays, "Habitat fub cute hominis fca-"biem cauffans, ubi veficulam excitavit, parum recedit, cor-"poris rugas fecutus, quiefcit iterum & titillationem excitat; "nudis oculis fub cuticula delitefcens obfervatur ab adfueto, "acu facile eximitur, ungui impofitus vix movetur, fi vero "oris calido halitu affletur, agilis in ungue curfitat.;" and Baron de Geer (Mem. pour fervir a l'Hiftoire des Infectes, Tom. VII.) obferves, that the infects he has had occafion to extract from itchy fores were extremely minute, not larger than a grain of common fand. "At firft," fays he, "when "they are taken from under the epidermis, they feem to be "without motion, but by degrees they begin to move their "feet, and to crawl, though flowly." — Editor.

 that that a friend of his at Hanover (who had the itch in a flight degree, and to whofe accurate inquiries with an excellent microfcope he acknowledges himfelf much indebted) found feveral infects in fuch furrows. Two of the longeft of the furrows were about an inch in extent. They feemed to be thoroughly dry, but exhibited here and there very minute fhining and tranfparent fpots. Thefe fpots, however, were not at all elevated above the furface of the fkin, and although feveral of them were opened and examined, no infect was found in them. Thefe furrows he has obferved only on the hands and fingers, having in vain fought for them on the legs, and other parts of the body, in his children, who had the itch in a high degree.

The appearance of thefe infects, when viewed through a microfcope, will be beft underftood by the figures of them which we have copied from Dr. Wichmann's work. The firft of thefe figures reprefents the acarus farinæ; the fecond and third are the itch infect, as they appeared through the author's microfcope; and the fourth is a figure of the fame infect, as given by Bonomo.

In order to affure himfelf of the accuracy of his figures, the author tells us he fent them, together with ſome veſicles taken from a patient with the itch, to his friend, Mr. Goetze, of Quedlinbourg, who has acquired diſtinguiſhed reputation as a naturaliſt, and whoſe ſkill in microſcopical obſervations is well known: this gentleman compared them with the ſame objects as ſeen through his own microſcope, and found them accurately repreſented.

From theſe figures, the body of the acarus farinæ appears to be more oblong than that of the itch inſect, and the feet of the latter, the author obſerves, are placed much nearer the head than in moſt other ſpecies of acari, and are ſhorter and thicker.

## Figures and Tables

**Fig. 1. 2. 3. 4. f1:**