# Errata

**Published:** 1788

**Authors:** 


E R R A T A.

Page 33 in the Note for '*c Vermiculorum*' read '*e Vermiculorum*'.

——41 —————'*incedentum*' ——— '*incedentem*'.

